# The effect of smear layer removal on *E. faecalis* leakage and bond strength of four resin-based root canal sealers

**DOI:** 10.1186/s12903-018-0655-7

**Published:** 2018-12-13

**Authors:** Laura Andriukaitiene, Xiaobo Song, Nannan Yang, Lippo V. J. Lassila, Pekka K. Vallittu, Eero Kerosuo

**Affiliations:** 1 Department of Clinical Dentistry, Faculty of Health Sciences, UiT The Arctic University of Norway, N-9037 Tromso, Norway; 2 Department of Medical Biology, Faculty of Health Sciences, UiT The Arctic University of Norway, Tromso, Norway; 3 Department of Community Medicine, Faculty of Health Sciences, UiT The Arctic University of Norway, Tromso, Norway; 4 Department of Biomaterials Science, Institute of Dentistry, University of Turku, Turku, Finland; 5 Welfare Division, Turku, Finland; 6 Present address: Vilnius, Vilnius, Lithuania

**Keywords:** Root canal sealers, Bond strength, Bacterial leakage, Smear layer

## Abstract

**Background:**

The aim of the study was to assess bacterial sealability and bonding ability of methacrylate-based Resilon (RS, SybronEndo), Endo Rez (ER, Ultradent Products Inc), and epoxy-based AH Plus (AH, Dentsply/DeTrey), MTA Fill Apex (MTAF, Angelus Soluções Odontológicas) root canal sealers, and the effect of the smear layer removal on the sealability.

**Methods:**

One hundred thirty root segments were instrumented up to apical size #60 and rinsed with 2.5% NaOCl. Half of the roots were rinsed with 5ml 17% EDTA to remove the smear layer. All the roots were filled with AH, ER, MTAF sealers and gutta-percha, or RS with Resilon cones. After storage at 37°C for 7 days the samples were mounted into bacterial leakage assay for 50 days.

Another 100 roots were instrumented and rinsed as described above, split longitudinally, cut into the cervical, middle and apical parts. The sealers were injected through the plastic mould on the dentin surface. After 7 days of incubation at 37°C, bond strength was tested using a notched-edge test fixture (Crosshead, Ultradent Products Inc.) and a universal testing machine (Lloyd Instruments).

**Results:**

AH revealed the longest mean time for bacterial resistance by 29.4 and 36.8 days (with and without smear layer, respectively) followed by RS (15.1 and 24.7 days, respectively). The difference between materials was significant (*p*<0.001).

Bond strength values ranged from 0.2± 0.1 to 3.5± 0.7 MPa and increased from the apical to the cervical third. In the apical third, AH showed the highest mean (SD) bond values 1.4 (0.4) MPa and 1.7 (0.6) MPa (with and without smear, respectively, followed by RS, 0.5 (0.1) MPa and 0.8 (0.1) MPa, respectively. The difference between materials was significant (*p*=0.001).

**Conclusion:**

The effect of the smear layer removal on the sealability was material-dependent.

**Electronic supplementary material:**

The online version of this article (10.1186/s12903-018-0655-7) contains supplementary material, which is available to authorized users.

## Background

The main cause of pulpal and periradicular pathosis is microorganisms and their by-products in the root canal system [1]. Root canal treatment aims to eliminate microbes from an infected root canal and to seal the canal system to prevent bacteria ingress from the oral cavity [2] and to entomb any residual bacteria [3].

Ørstavik et al. [4] postulated that a correlation should exist between adhesive properties of a root canal sealer and its sealability. Although methacrylate-based sealers Endo Rez (ER, Ultradent Products Inc., South Jordan, UT) and Epiphany/ Resilon (RS, SybronEndo, Orange, CA) produce longer and more frequent resin tags to dentinal tubules, compared to epoxy resin-based AH Plus (AH, Dentsply/ DeTrey, Konstanz, Germany), the latter has shown significantly higher bond strength values [5]. Studies reporting both bond and sealability are sparse. Eldeniz et al [5, 6] found no significant difference in the sealability between AH and ER, although AH had three-times higher bond strength. Similar lack of correlation between bond and sealability has been shown with AH, compared to a salicylate-based sealer [7, 8].

In order to enable disinfectants to reach the bacteria from the dentinal tubules, the removal of the smear layer before intracanal medication and filling of root canals is widely advocated [9]. The final rinse of root canal with NaOCl and 17% EDTA was confirmed as the most effective combination in the removal of the smear layer [10]. This was supposed to increase the adhesion and sealability of a filing [11]. But in contrast, Saleh et al. [7] found that EDTA did not increase the bond strength of any tested materials, including AH. Moreover, the same research group in another study found that the use of EDTA did not improve the sealability of any of the tested material, including AH and RS [8].

The aims of this study were therefore to assess the bacterial sealability and bonding ability of two methacrylate resin-based root canal sealers, ER and RS, and two epoxy-based sealers, AH and MTA Fill Apex (MTAF) (Angelus Soluções Odontológicas, Londrina, PR, Brazil), and to test the hypothesis that the removal of the smear layer would improve the sealability.

## Materials and Methods

### Tested materials

Two methacrylate resin-based sealers Endo Rez (ER, Ultradent Products Inc., South Jordan, UT) and Epiphany/ Resilon (RS, SybronEndo, Orange, CA) and two epoxy-based sealers AH Plus (AH, Dentsply/ DeTrey, Konstanz, Germany) and MTA Fill Apex (MTAF) (Angelus Soluções Odontológicas, Londrina, PR, Brazil) were used in this study.

### Selection of the teeth

A total of 230 human maxillary central incisors extracted for reason not related to this study were used. Periapical radiographs (Bel-Ray II AC, Belmont Equipment, Somerset, NJ, USA) were taken in mesio-distal and bucco-lingual planes to exclude severe root canal calcification, apical curvatures, or any resorptive alteration of the canal lumen. All the teeth were subject to surface disinfection by immersion in 0.5% Chloramine T, followed by removal of all adhering soft tissues and debris by scaling, washed under running tap water, placed in distilled water, and refrigerated at 4C° for 24 hours before use.

### Preparation of the samples for bacterial leakage assay

The crowns of 130 teeth were removed with a diamond bur in a high-speed hand piece under water-cooling, leaving 10mm of the root segment. All roots were inspected for the presence of cracks with a stereomicroscope under x40 magnification. ProTaper Universal NiTi rotary files (Dentsply/Maillefer, Ballaigues, Switzerland) were used to prepare each root canal to size #50 and stainless steel K-files (Dentsply/Maillefer) to finish preparation up to size #60. The root canals were irrigated with 2.5% 3ml NaOCl after the use of each file. All the roots were randomly divided into two groups. Half of instrumented roots were rinsed with 5ml of 17% EDTA for 5 min to remove the smear layer [12, 13]. Distilled water was finally used to rinse the roots thoroughly. All the roots were autoclaved at 121+/- 2°C for 20 min. After sterilization, the root canals were dried with sterile paper points (Dentsply/Maillefer). All the roots with and without the smear layer were assigned to eight experimental subgroups (n=15) and two control groups (n=5) as shown in Table 1. All the tested sealers were mixed according to manufacturer’s instructions and applied into the root canals on the master gutta-percha (GP) cone size #60 (Dentsply/Maillefer) or Resilon cones (SybronEndo), respectively. The canals were obturated with the lateral condensation method. Additional GP cones size A (Dentsply/Maillefer) were placed after lateral compression with same size spreader until the cervical part of the root was filled. Eventually excess GP from the coronal part was removed with a heated hand plugger and condensed vertically. Aseptic techniques were employed throughout the procedure. The specimens were kept in sealed tubes with sterile water at 37°C for 7 days to allow the sealers to set. The positive controls prepared and rinsed with EDTA as described above were obturated without a sealer (core material only), simulating poor obturation.

**Table 1:** The tested materials, their codes, and the number of specimens in each group for the bacterial leakage and bond strength experiments

Material	Code	Experiment			
		Leakage (n)	Bond (n)		
			Apical	Middle	Cervical
AH Plus/GP w/ EDTA	AH-ns	15	8	8	9
AH Plus/GP w/o EDTA	AH-s	15	8	10	10
EndoRez/ w/ EDTA	ER-ns	15	7	8	8
EndoRez/GP w/o EDTA	ER-s	15	7	7	8
RealSeal/Resilon w/ EDTA	RS-ns	15	9	8	9
RealSeal/Resilon w/o EDTA	RS-s	15	9	8	9
MTA Fill Apex/GP w/ EDTA	MTAF-ns	15	8	9	8
MTA Fill Apex/GP w/o EDTA	MTAF-s	15	7	7	7
Composite w/ EDTA	Composite-ns	-	10	9	10
Composite w/o EDTA	Composite-s	-	9	9	10
Positive control w/ EDTA	PC	5^a^	30^b^		
Negative control w/o EDTA	NC	5^c^			

*n* number of specimens, *GP* gutta-percha, *w/* with, *w/o* without, *ns* smear layer removed, *s* smear layer left *in situ*

^a^Root canals obturated with core material only, simulating poor obturation

^b^Tested materials were applied on every third crown with class III standard cavity as described above

^c^No root canal filling, just sealing coronal and apical part of the root with the sticky wax

### Bacterial leakage assay

The two-chamber microleakage device [8, 14] was used with minor modifications. The specimens were inserted through a cut tip of 15ml polyethylene tubes (upper chambers), leaving 3mm of the cervical part inside the tube and the remaining part hanging out of it. Melted sticky wax was first applied on the outer surface of the 3mm cervical part, leaving the surface with the canal orifice exposed in the experimental and positive control groups, but fully covering it in the negative control group. Thereafter the wax was applied on the hanging part of the root and the tube interface, leaving 3mm of the apical part uncovered similarly in all the groups. These mounts were then tightly sealed with sticky wax to sterile 50 ml polyethylene tubes (lower chambers) containing 8 ml of sterile Trypticase Soy Broth (TSB; Oxoid Ltd, Basingstoke, UK). The apices extruding from the upper chambers were hanging vertically 2mm in the broth.

*Enterococcus faecalis* ATCC 29212 was used as the test strain. After growing in TSB at 37°C overnight, 3ml of the overnight bacterial culture was added to each upper chamber. The mounts were incubated at 37°C for 50 days. The bacterial culture in the upper chamber was replaced with fresh bacterial every second day to maintain bacterial sufficiency and viability. The bottom chambers of all the mounts were checked every second day for turbidity, the evidence for bacterial penetration along the root canal filling.

On observation of turbidity, the seal was broken and the bacterial cultures were then streaked on Trypticase Soy Agar (TSA; EMD Millipore Corporation, Billerica, MA, USA) plates for colony morphology observation and PCR identification. The bacteria growing on 25 plates, randomly chosen from the eight experimental subgroups, were identified by PCR assay with species-specific primers targeting *E. faecalis* 16S rRNA [15]. The date of leakage was recorded for each leaking sample.

### Preparation of the samples for bond testing

The method previously described by Jessop [16] was used with minor modifications for the preparation and testing of the samples.

One hundred teeth were decoronated at the cement-enamel junction using a slow speed diamond-watering blade (Ernst Leitz GmbH, Wetzlar, Germany), split longitudinally in the bucco-lingual direction and inspected for presence of cracks with a stereomicroscope under x40 magnification, grounded on a water-irrigated grinding wheel (Struers LaboPol-21) until smooth and flat surface, using 2000-grit (FEPA) silicon carbide paper and cut into three parts: cervical, middle, and apical. Each specimen was fixed in the acrylic resin (Heraeus Kulzer Dental GmbH, Laboratory Products Division, Hanau, Germany). All the roots were divided into two groups according to the final exposed dentin surface treatment. In group A, the smear layer was removed by rinsing each specimen with 3ml 2.5%NaOCl solution for 1min, followed by 3ml 17%EDTA for 1min. Group B specimens were irrigated for 1min only with 3ml 2.5% NaOCl.

Each group was divided into five subgroups: i) AH was injected into the plastic mould, with 2.4 mm diameter and 2mm cylindrical button height (Ultradent Products Inc. South Jordan, UT, USA) on the root dentin bonding surface, ii) RS was similarly applied and light-cured for 40 sec according to manufacturer’s instructions, iii) MTAF and iv) ER were applied as AH, v) composite specimens were prepared according to manufacturer’s instructions by applying 3M ESPE Scotchbond Etchant, Adhesive and Primer (3M Dental Products, St. Paul, MN, USA) on the dentin specimens. Finally, the composite 3M ESPE Filtec Supreme XP was packed through the mould on the bonding surface and light-cured for 20 sec. All the samples were left for 8 hours in a water bath at the room temperature and the jig was removed. Afterwards they were placed in an incubator at 37°C and 100% humidity for 7 days. For comparison, every third crown was used for testing the dentin bonding to simulate a restorative procedure in a class III standard cavity (1.5 mm deep).

### Bond strength testing

The specimens were tested using a notched-edge shear test fixture (Crosshead, Ultradent Products Inc.) on a universal testing machine (Lloyd LRX; Lloyd Instruments, Fareham Hants, UK) and the results expressed in MPa by diving the force needed to break the bond (N) by the surface area in mm^2^. Failure modes obtained by the shear-bond testing were reported and a mean and standard deviation calculated.

### Statistical Analysis

The group size of 15 was considered appropriate in this *ex vivo* study. Too limited or inconsistent data in the previous literature does not allow to estimate a golden standard to which the other materials can be compared.

In the leakage assay, the Kaplan Meier test for survival analysis was used. The median time of leakage was calculated and pairwise comparisons of groups were performed by using the log-rank test. Bond strength between the groups was analysed using the Two-way ANOVA with Tukey’s post hoc test. Data were entered and analysed by the statistical program package IBM SPSS statistics 21.0 (IBM, Somers, New York, NY, USA).

## Results

### Bacterial leakage assay

**Fig. 1:**
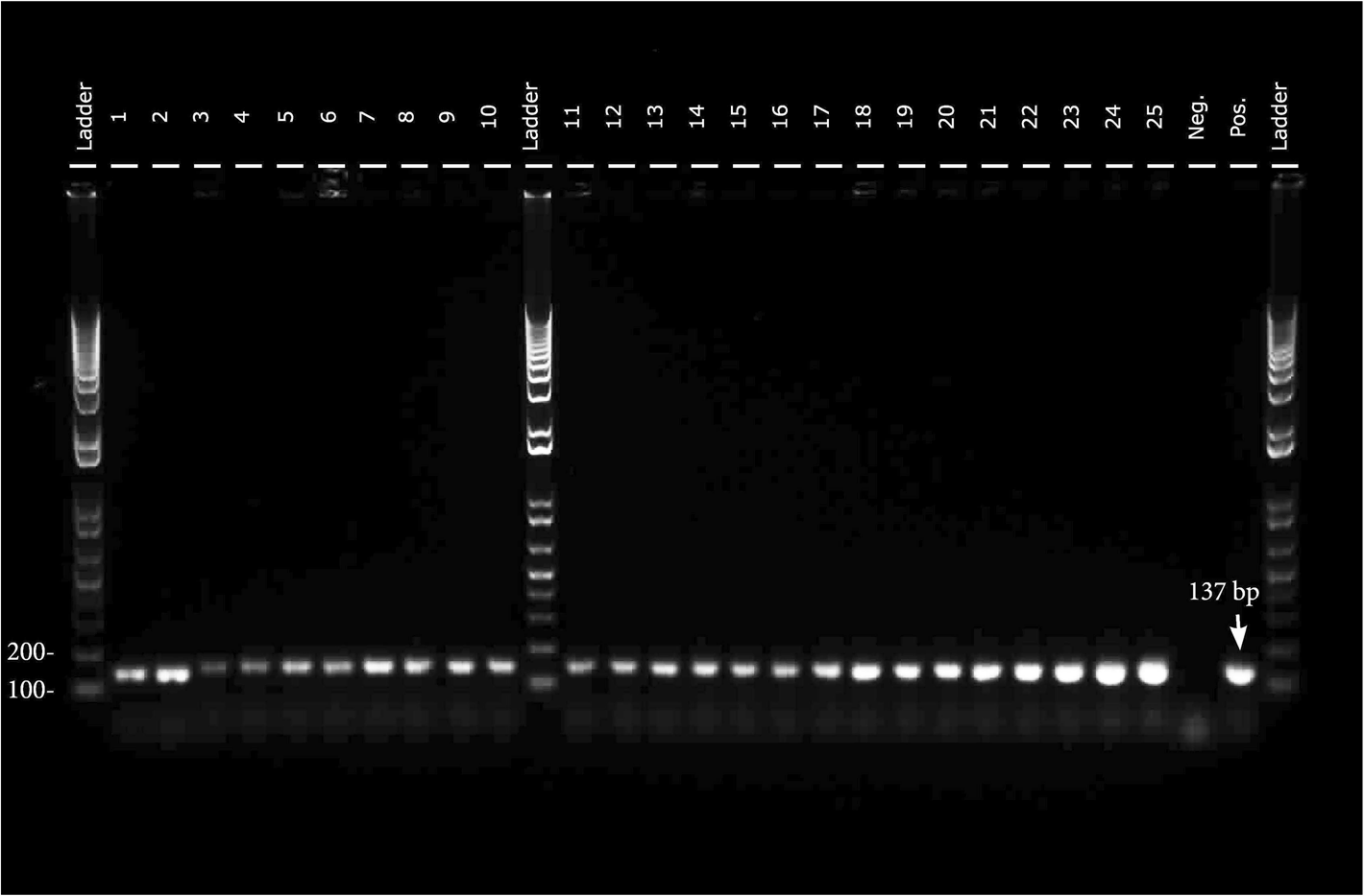
PCR profiles of 16S rRNA gene from the bacterial samples in lower chamber. The lanes of ladder are 1 kb plus ladder; the lanes of 1-25 are samples. Neg, the lane of negative control; Pos, the lane of positive control of *E. faecalis* ATCC 29212 at the size of 137 bp

All the cell colonies on TSA plates appeared as small, smooth, cream or white colonies with entire edges and were identical to those of *E. faecalis* ATCC 29212*.* The PCR assay revealed 23 samples showing one band at the same size as 137 bp of positive control of *E. faecalis* ATCC 29212 and two samples showing one band a bit lower than the positive control (Fig. 1). Further DNA sequencing confirmed that these two strains were also *E. faecalis* ATCC 29212 having 99-100% sequence identity.

All the samples leaked within 41 days (range 6-41 days), except the negative controls (*N*=5) which remained without leakage throughout the 50 days test period. All the positive controls (*N*=5) samples leaked on the second day (Table 2).

**Table 2:** Proportion of leaked samples and the number of days the samples resisted the bacterial leakage, expressed as mean, range, and standard deviation (SD). Four group codes, see Table 1

Material/smear	P	Mean	Range	SD	D
AH-s	15/15	29.4	23-35	1.0	b
AH-ns	15/15	36.8	30-41	1.1	a
ER-s	15/15	11.8	9-16	0.7	e
ER-ns	15/15	8.7	6-13	0.5	d
RS-s	15/15	24.7	20-37	1.1	b
RS-ns	15/15	15.1	11-20	0.8	c
MTAF-s	15/15	8.9	6-13	0.7	d
MTAF-ns	15/15	13.3	9-18	0.8	c
Positive control	5/5	2	2	0	
Negative control	0/5	50	50	0	

*AH* AHPlus, *ER* EndoRez, *RS* RealSeal/Resilon, *MTAF* MTA Fill Apex, *s* smear layer in situ

*ns* smear layer removed with EDTA, *P* proportion of samples leaked, *Mean* mean time of leakage days, *Range* range of leakage days, *SD* Standard deviation of the leakage days, *D* Log-rank test (*P*< 0.05): experimental groups with different letter are significantly different from each other

**Fig. 2:**
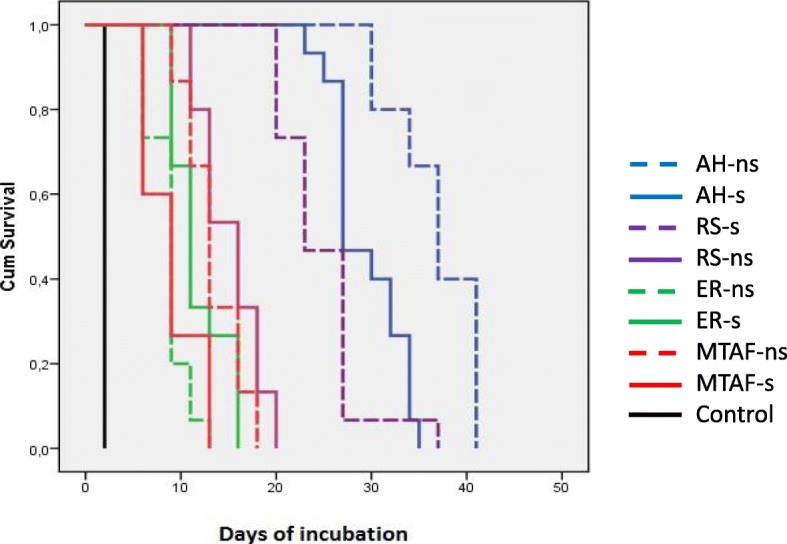
Kaplan Meier cumulative survival curves of samples expressed in number of incubation days resisting the bacterial leakage. Solid line, smear layer left *in situ*; dotted line, smear layer removed with EDTA. None of the negative control samples leaked in 50 days

The Kaplan-Meir survival curves showed that the AH-ns group significantly differed from all the other groups (*p*<0.001; Mantel-Cox Chi-square analysis) (Fig. 2).

### Bond strength test

All the tested sealer materials showed an increase in bond strength when going from the apical region towards the crown. Bonding to the crown dentin after the removal of the smear layer with composite resin, used as a reference material, showed the highest strength values, 13.7±1.5 MPa (Table 3). The strength values of the sealers bonded to the root canal wall dentine ranged from 0.2± 0.1 to 3.5± 0.7 MPa, depending on the material, location or removal of the smear layer with EDTA (Table 3). The removal of the smear layer increased the bond strength of all the sealers in all thirds of the root, except in the AH group in the cervical and middle third of the root and in the MTAF group in the cervical third, but none of the differences were statistically significant when tested by Tukey’s pairwise comparisons (*P*>0.05) (Table 3). In all the tested root dentin sites, the composite resin used for the reference showed significantly higher (*P*<0.001) bonding values (range from 3.1±0.5 to 10.8±1.9 MPa) compared to any of the sealers, irrespective of the site of the tooth or the smear layer removal (Table 3). In the apical third, AH showed highest bond strength from all the tested sealers, and differed significantly from all other sealers groups (AH-ns vs. RS-ns *p*=0.001, Tukey’s pairwise comparisons), regardless of the smear layer removal (Table 3).

**Table 3:** Mean ± standard deviation (SD) of shear-bond strength values (MPa) of four endodontic sealer materials and a composite resin filling material tested on four different dentin sites, the crown part of the tooth and apical, middle and cervical thirds of the root canal wall, with and without removing the smear layer using EDTA

Material	Apical	Middle	Cervical	Crown	*P*-value
	Mean (SD)	Mean (SD)	Mean (SD)	Mean (SD)	(sites)
AH-s	1.4(0.4) N=8xa	2.0(0.8) N=10xa	3.5(0.7) N=10xb	3.4(0.4) N=3xb	*P*<0.001
AH-ns	1.7(0.6) N=8xa	1.9(0.4) N=8xa	3.0(0.5) N=9xb	5.7(0.5) N=3xb	*P*<0.001
ER-s	0.2(0.1) N=7ya	0.2(0.1) N=7ya	0.4(0.1) N=8yb	0.7(0.2) N=2yc	*P*<0.001
ER-ns	0.4(0.1) N=7ya	0.5(0.4) N=8ya	0.1(0.4) N=8yb	0.9(0.1) N=3yb	*P*=0.005
RS-s	0.5(0.1) N=9y	0.8(0.1) N=8x	1.0(0.6) N=9y	1.2(0.1) N=3y	*P*=0.360
RS-ns	0.8(0.1) N=9ya	1.0(0.3) N=8xa	1.7(0.5) N=9yb	2.6(0.4) N=3yc	*P*<0.001
MTAF-s	0.2(0.1) N=7ya	0.4(0.1) N=7ya	1.1(0.3) N=7yb	0.9(0.1) N=2yb	*P*<0.001
MTAF-ns	0.3(0.1) N=8ya	0.5(0.1) N=9yb	1.0(0.3) N=8yc	1.1(0.2) N=3yc	*P*<0.001
Comp-s	3.1(0.5) N=9za	4.8(1.3) N=9za	9.6(2.1) N=10zb	11.7(1.2) N=3zb	*P*<0.001
Comp-ns	3.4(0.9) N=9za	5.5(0.8) N=9zb	10.8(1.9) N=10zc	0.7(1.5) N=3zd	*P*<0.001
*P*-values (material groups)	*P*<0.001	*P*<0.001	*P*<0.001		

*AH* AHPlus, *ER* EndoRez, *RS* RealSeal/Resilon, *MTAF* MTA Fill Apex, *Comp* Composite, *s* smear layer *in situ*, *ns* smear layer removed with EDTA, *N* number of samples

a-d, pairwise comparison between the dentin sites, Tukey’s test

x-z, pairwise comparisons between the material groups, Tukey’s test

### Relationship between shear bond strength and bacterial leakage

**Fig. 3:**
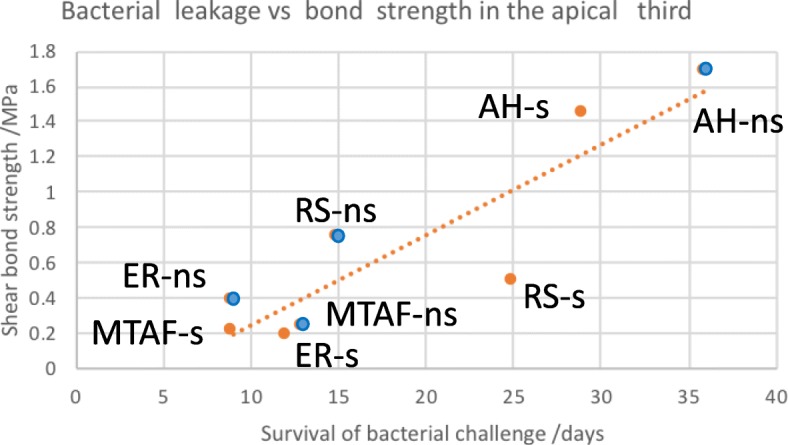
Survival of the bacterial challenge (mean number of days resisting the leakage) of *E. faecalis* strain in a two-chamber model in relation to the mean of the shear bond strength (MPa) in the apical third of the root in the presence (red dots) and absence (blue dots) of the smear layer. Dotted line represents the trend. AH, AH Plus/gutta-percha; RS, RealSeal/Resilon; ER, EndoRez/gutta-percha; MTAF, MTA Fill Apex/gutta-percha

There was an obvious overall trend that higher bond strength values resulted in less bacterial leakage (Fig. 3). In the apical third, the removal of the smear layer increased bond strength within all the materials tested, but had a favourable effect on bacterial sealability with two materials only, AH and MTAF.

## Discussion

There are numerous of studies addressed to test the bond strength separately from the coronal or apical bacterial leakage of the endodontic sealer cements. However, to our knowledge there is no study that could assess the bond strength of the sealer and its resistance to the microbial leakage. We tested four sealer cements that are commonly used in the clinical practice. AH Plus is considered to be as the golden standard. It has very favorable physical properties and it has been widely tested [17]. EndoRez features long resin tags into dentinal tubules that might be beneficial for the mechanical retention to the root canal dentin [18]. Several bacterial leakage studies reported conflicting findings between AH Plus and EndoRez [19–23], therefore we tested both materials as they are still commonly used in clinical practice. Epiphany (RealSeal) and MTA Fill Apex are relatively new materials. Epiphany has been claimed to have an ability to bond to the dentin wall and the core material and to form a gap-free solid monobloc, while MTA Flill Apex should exhibit MTA features. Two different irrigation regimes were tested to explore a possible influence of the smear layer to the adhesion as well as the bacterial leakage and to assess those important parameters.

Dye and isotope penetration, glucose and fluid infiltration are suggested to assess the coronal leakage [24], but the two-chamber bacterial method may best simulate the clinical conditions [8, 14, 25], hence used in this study. Rechenberg et al. [26] recently challenged the validity of the two-chamber model, as they found bacterial penetration between sealing wax and the root interface in both the experimental and control groups after 56 days. In contrast, our study showed clear differences in the experimental groups within 50 days, although the root parts were similarly covered by wax in each group. They also pointed out improper sample preparation for the control groups. In our study, we used melted pre-autoclaved sticky wax to fix samples to upper chamber tubes first from the coronal aspect to assure close contact to the root and the tube. As suggested by De-Deus [27], we paid attention to a similar design of the groups by leaving the root apices uncovered for all the groups, including the positive and negative controls.

To overcome possible variations in root canal anatomy, we used upper incisors only. Moreover, their anatomy was examined using radiographs in two planes to exclude those with severe root canal calcification or fracture lines, or any resorptive alteration of the canal lumen.

*E. faecalis* was chosen as the test bacterium, because it is frequently recovered in persistent periradicular lesions [3, 15, 28]. It shows an ability to penetrate dentin tubules, form biofilms on biotic/abiotic surfaces, endure prolonged nutritional deprivation, resist intracanal medication and produce virulence factors that cause persistent infection and periradicular inflammation, such as lytic enzymes, cytolysin, aggregation substance, pheromones, and lipoteichoic acid [29, 30].

None of the adhesion tests have been generally accepted as standard. Adhesion tests measure either tensile bond strength, where the bond is broken by a force perpendicular to the interface between material and surface, or shear strength , where a force is parallel to the interface between material and surface. The shear test was developed to measure the bond of endodontic sealers to root dentin and it proves to be effective and reproducible [31]. The tensile bond strength test was used by Saleh et al. [7], to assess sealer materials. But we rather wanted to use a modified bond test by Jessop [16], where shear stress is predominant. The shear bond reflects a clinical scenario adequately because the forces simulate closely the ones that may distort the obturation e.g. during the dowel space preparation [31].

AH resisted the bacterial penetration the longest time, regardless of the pre-treatment of the dentine with EDTA. This finding is in contrast to the results of Saleh et al [8], who found AH to leak more than RealSeal sealer/cones (later called RS/ Resilon), when EDTA was used to remove the smear layer. Both studies used the two-chamber test model modified from Torabinejad et al [14] and *E. faecalis* as the test bacterium, but different obturation techniques. Opening the dentinal tubules with EDTA may have favoured more the lateral compaction used in our study, compared to the single cone technique used by Saleh et al. [8], although the relationship between sealing ability of endodontic sealers and their penetration in dentinal tubules has not been confirmed [32]. When *S. mutans* was used as the test bacterium, Shipper et al [25] found significantly less leakage with Resilon/Epiphany compared to GP/AHPlus, but no difference when Epiphany was used with GP. In our study, both MTAF and ER leaked considerably more than AH, irrespectively of the removal of the smear layer. For ER, this was in contrast to the findings of Eldeniz & Ørstavik [6], who did not find any difference in leakage between AH and ER. This may be explained by the different test microbes used, *S. mutans* vs. *E. faecalis.*

In our study, the removal of the smear layer significantly improved the resistance against bacterial leakage in the roots filled with GP/AH and GP/MTAF, but deteriorated the seal for RS and ER. For AH, this contrasts with the previous findings by Saleh et al. [8], but confirms their results with RS. Traditionally, smear layer is regarded to be removed, as it may be infected [33, 34] and may act as barrier to obstruct access of medicaments to the bacteria deeper in the dentinal tubules [35, 36]. Although this view has been challenged [37], the removal of the smear layer is still widely advocated in textbooks [9]. The penetration of a sealer into open dentinal tubules is suggested to improve the sealing ability by mechanical interlocking [38]. Potential antimicrobial activity of resin tags inside dentinal tubules may also hamper the colonization of residual bacteria and reinfection of a root canal [39, 40] RS is shown to penetrate deeper into the dentinal tubules than AH, while ER showed the lowest penetration ability [18, 41]. On the other hand, recent studies have found no statistical difference in tag penetration between AH and MTAF [42, 43]. However, our bond strength and microleakage results do not favour RS, despite of its ability to form the longest tags. Nevertheless, the smear layer removal improved AH bonding and sealing abilities, indicating that when AH is used as a sealer, EDTA could also be used safely in vital cases and thus be part of a routine in all endodontic obturations.

In the present study, AH significantly reduced the enterococcal leakage in comparison with the other root canal sealers tested. This could be due to sealability of the material only, and/or due to its antimicrobial properties. A number of studies have demonstrated that AH has significant antimicrobial activity against *E. faecalis* and other organisms in root canal. Saleh et al. [12] stated that AH killed all bacteria in the dentine tubules within the zone of 300 mm around root canal. Kayeoglu et al. [44] found that an epoxy resin-based AH effectively reduced colony forming units of *E. faecalis.* Heyder et al. [45] revealed that AH had an antibacterial effect on three species, *E. faecalis*, *F. nucleatum* and *P. gingivalis*, while ER and ProRoot MTA displayed no suppressive effect on *E. faecalis.* To the contrary, Baer & Maki [46] failed to show the inhibition activity on the growth of *E. faecalis* of AH and RS.

Regarding sealability, our results are in line with those by Baechtold et al. [47], who found AH to present high adhesion/bond strength to root canal wall and root filling materials. Our result in the apical third are in line with those of Eldeniz et al. [5], who found the removal of the smear layer to increase the bond of AH and RS, although the site of the root was not specified in their study. In contrast, we found an opposite trend with AH in the middle and cervical thirds.

We found higher bond strength values for RS, regardless of the smear removal, as compared with the results of Wachlarowicz et al. [48]. However, they used a different modification of the test model and did not specify whether the bonding substrate was the root canal or the outer root dentin, which might influence the results. In contrast to a recent study by Haragushiku et al. [49], all the tested materials showed decreasing bond strength from the cervical to apical region. But the finding is in line with the previous studies that showed differences in the composition of dentin properties, partly due to the distribution of dentinal tubules [50, 51].

There was a clear trend that higher bond would also indicate less leakage, although in that respect ER and RS acted differently when the effect of the smear removal was concerned. The mechanism, behind better sealing capacity of materials with better bonding properties to dentine, relates to the capability of bonding interfaces to resist stress of the curing contraction of the sealer. From the clinical point of view, our results will favour AH, as it would be desirable to select a sealer material that apart from justified sealing ability, would also resist any disturbing mechanical forces e.g. during the dowel space preparation.

## Conclusions

As a conclusion, the present study showed that among the four resin-based sealers tested, the effect of the smear layer removal on the sealability was material-dependent. AH showed the highest bond and lowest bacterial leakage. As the removal of the smear layer improved both the bond and the sealability for AH, the results suggest the clinical use of EDTA for root canal conditioning also in vital cases, if AH is used.

## Additional file

Additional file 1: Legislation. (DOCX 16 kb)

## Abbreviations

AH: AHPlus
ER: EndoRez
GP: Gutta- percha
MTAF: MTA Fill Apex
n: Number of specimens
ns: Smear layer removed
RS: Resilon
s: Smear layer left *in situ*
SD: Standard deviation
w/: with
w/o: without
