# Supplementary material for: The effect of smear layer removal on E. faecalis leakage and bond strength of four resin-based root canal sealers
Source: BMC Oral Health. 2018 Dec 13;18:213. doi: 10.1186/s12903-018-0655-7 (PMC6293555; doi:10.1186/s12903-018-0655-7)
Supplement: Supplementary file 1 — Legislation. (DOCX 16 kb) [file 12903_2018_655_MOESM1_ESM.docx]

N.B Unofficial translation, legally binding only in Finnish and Swedish

**MINISTRY OF SOCIAL AFFAIRS AND HEALTH, FINLAND**

**No. 101/2001**

**Act on the Medical Use of Human Organs, Tissues and Cells**

Issued in Helsinki, on 2nd February 2001

Section 20 (689/2012)

*Change in purpose for which tissue samples will be used*

Tissue samples taken for therapeutic or diagnostic purposes may be surrendered and used for medical research with the patient’s consent. If the person is under-age or incapacitated, consent must be obtained from his or her legal representative. If it is impossible to obtain the person’s consent because the person has died, the samples can be used for medical research or transferred to a biobank referred to in the Biobank Act after the ethics committee referred to in the Medical Research Act has issued a favourable opinion on the matter. If the opinion of the ethics committee is negative, the National Supervisory Authority for Welfare and Health shall issue a decision on the matter upon application. If there is reason to assume that the person while alive would have objected to use of his samples for research purposes, the sample may not be transferred to a biobank. Further preconditions for transfer and handling of samples are laid down in the Biobank Act.

Tissue samples taken for therapeutic or diagnostic purposes, or to establish the cause of death, may be surrendered and used for medical research, method development, quality management and teaching purposes with permission from the health care unit or other unit for whose activities the sample was taken, as long as no personal data are used in the surrender or use situation.

Tissue samples taken for therapeutic, diagnostic or medical research purposes may be surrendered and used to establish the hereditary character of a disease diagnosed in another person only if the person from whom the sample was taken gives his or her consent. If the person is under-age or incapacitated, consent must be obtained from his or her legal representative. If the person has died or the tissue sample was taken in order to establish the cause of death, the health care unit or other unit for whose activities the sample was taken shall decide whether to surrender the tissue sample, unless the deceased specifically prohibited this while alive.

Samples taken for therapeutic or diagnostic purposes, to establish the cause of death, or for medical research can be surrendered in order to establish the identity of biological parents to a research institution specified by a court of law or some other authority or to identify a deceased person to the police.

Tissue samples taken for therapeutic or diagnostic purposes, or to establish the cause of death, may not be surrendered or used for any purpose other than that for which they were taken if this will hamper their use for the original purpose.

**2.2.2001/101**

**Laki ihmisen elimien, kudoksien ja solujen lääketieteellisestä käytöstä**

**20 §** [**(30.11.2012/689)**](http://www.finlex.fi/fi/laki/ajantasa/2001/20010101#a30.11.2012-689)

**Kudosnäytteiden muuttunut käyttötarkoitus**

Hoidon tai taudinmäärityksen vuoksi otettuja kudosnäytteitä saa luovuttaa ja käyttää lääketieteelliseen tutkimukseen potilaan suostumuksella. Jos henkilö on alaikäinen tai vajaakykyinen, suostumus tulee saada hänen lailliselta edustajaltaan. Jos suostumusta henkilön kuoleman johdosta ei ole mahdollista hankkia, näytteitä voi käyttää lääketieteelliseen tutkimukseen tai siirtää biopankkilaissa tarkoitettuun biopankkiin lääketieteellisestä tutkimuksesta annetussa laissa tarkoitetun eettisen toimikunnan annettua asiasta myönteisen lausunnon. Jos eettisen toimikunnan lausunto on kielteinen, Sosiaali- ja terveysalan lupa- ja valvontavirasto tekee hakemuksesta asiasta päätöksen. Jos on syytä olettaa, että henkilö eläessään olisi vastustanut näytteidensä tutkimuksellista käyttöä, näytettä ei saa siirtää biopankkiin. Näytteiden siirtämisen ja käsittelyn edellytyksistä säädetään lisäksi biopankkilaissa.

Hoidon, taudinmäärityksen tai kuolemansyyn selvittämisen vuoksi otettuja näytteitä voidaan luovuttaa ja käyttää lääketieteelliseen tutkimukseen, menetelmäkehitykseen, laadunhallintaan ja opetukseen sen terveydenhuollon toimintayksikön tai muun yksikön luvalla, jonka toimintaa varten näyte on otettu, jos näytteitä luovutettaessa tai käytettäessä ei käsitellä henkilötietoja.

Hoidon, taudinmäärityksen tai lääketieteellisen tutkimuksen vuoksi otettuja kudosnäytteitä saadaan luovuttaa ja käyttää toisen ihmisen todetun sairauden perinnöllisyyden selvittämiseen vain, jos se, josta näyte on otettu, antaa siihen suostumuksensa. Jos henkilö on alaikäinen tai vajaakykyinen, suostumus tulee saada hänen lailliselta edustajaltaan. Jos henkilö on kuollut tai kyse on kuolemansyyn selvittämisen vuoksi otetusta kudosnäytteestä, kudosnäytteen luovuttamisesta päättää se terveydenhuollon toimintayksikkö tai muu yksikkö, jonka toimintaa varten kudosnäyte on otettu, jollei vainaja ole tätä eläessään kieltänyt.

Hoidon, taudinmäärityksen, kuolemansyyn selvittämisen tai lääketieteellisen tutkimuksen vuoksi otettuja näytteitä voidaan luovuttaa biologisten vanhempien selvittämistä varten tuomioistuimen tai muun viranomaisen määräämälle tutkimuslaitokselle tai kuolleen tunnistamista varten poliisiviranomaiselle.

Hoidon, taudinmäärityksen tai kuolemansyyn selvittämisen vuoksi otettuja kudosnäytteitä ei saa luovuttaa tai käyttää muuhun tarkoitukseen kuin mihin ne on otettu, jos se haittaa kudosnäytteiden alkuperäisen käyttötarkoituksen toteuttamista.
